# Sheep Feed and Scrapie, France

**DOI:** 10.3201/eid1108.041223

**Published:** 2005-08

**Authors:** Sandrine Philippe, Christian Ducrot, Pascal Roy, Laurent Remontet, Nathalie Jarrige, Didier Calavas

**Affiliations:** * Agence Française de Sécurité Sanitaire des Aliments, Lyon, France;; † Institut National de la Recherche Agronomique, Theix, France;; ‡ Centre Hospitalo-Universitaire Lyon-Sud, Lyon, France

**Keywords:** Scrapie, Sheep, Transmission, Epidemiology, Case-Control studies, Risk Factors, France, Transmissible Spongiform Encephalopathy

## Abstract

Scrapie is a small ruminant, transmissible spongiform encephalopathy (TSE). Although in the past scrapie has not been considered a zoonosis, the emergence of bovine spongiform encephalopathy, transmissible to humans and experimentally to sheep, indicates that risk exists for small ruminant TSEs in humans. To identify the risk factors for introducing scrapie into sheep flocks, a case-control study was conducted in France from 1999 to 2000. Ninety-four case and 350 control flocks were matched by location and main breed. Three main hypotheses were tested: direct contact between flocks, indirect environmental contact, and foodborne risk. Statistical analysis was performed by using adjusted generalized linear models with the complementary log-log link function, considering flock size as an offset. A notable effect of using proprietary concentrates and milk replacers was observed. The risk was heterogeneous among feed factories. Contacts between flocks were not shown to be a risk factor.

## Abstract 2

Proprietary concentrates and milk replacers were linked to risk for scrapie.

Scrapie is a transmissible spongiform encephalopathy (TSE) affecting sheep and goats (1), as is Creutzfeldt-Jakob disease (CJD) in humans or bovine spongiform encephalopathy (BSE) in cattle. Moreover, scrapie is contagious in natural conditions (2). Though genetic determinism is a major feature of scrapie, the infectious agent is nonetheless needed for the disease to develop (3,4).

Known to exist for centuries, scrapie was thought to be a possible origin of BSE, although this hypothesis has not yet been verified. Sheep and goats can be experimentally infected with BSE, resulting in a disease that is impossible to distinguish from natural scrapie (5). Since BSE is implicated in the emergence of variant CJD (6,7), the existence of BSE in small ruminants poses a further risk for human health. Scrapie has become a public health challenge, and its propagation must be stopped; therefore, the risk factors for the introduction of scrapie in sheep must be understood.

In sheep infected with scrapie, the infectious agent is widely distributed in the organism. In particular, the gut-associated lymphoid tissues and the placenta are considered highly important in spreading the disease (8) and can contaminate the environment (9). Because feed is considered to be the main, if not the only, contamination source of BSE in cattle (10,11), it can also be presumed to be a potential risk factor for scrapie in sheep.

A case-control study of infected and scrapie-free flocks was conducted to identify risk factors for introducing scrapie into sheep flocks in France. Various risk factors hypotheses were tested from the most plausible to the weakest.

## Materials and Methods

### Study Design

A case-control study of infected and scrapie–free flocks was designed (Appendix). A flock was defined as having at least 20 adult ewes. To consider the heterogeneity of exposure to scrapie risk, cases and controls were matched according to main sheep breed and location. A "case" was any flock having ≥1 animal that had been shown as scrapie-positive by the French surveillance network from January 1996 to July 2000 (12). Four frequency-matched control flocks were randomly selected from the sheep flocks in which scrapie had never been reported. Flocks that did not meet this criterion were excluded.

The suspected risk factors were grouped into 3 categories corresponding to the main working hypotheses of scrapie dissemination. The first category covered risks for transmission by direct contact between flocks and indirectly through the environment. The second category covered foodborne risks. The third category covered other environmental dissemination risks such as equipment sharing between farms or transmission through hay mites. Table 1 describes the 22 potential risk factors studied.

**Table 1:** Univariate analysis of potential risk factors

Risk factors	Modalities	No. controls (%)	No. cases (%)	Univariate Clog-log model	
				OR*	80% CI
Direct contacts between flocks and indirect environmental contacts					
Purchase of ewes	No	227 (65)	55 (59)	1.0	–
	Yes	123 (35)	39 (41)	1.3	1.0–1.8
Purchase of rams	No	146 (42)	33 (35)	1.0	–
	Yes	204 (58)	61 (65)	1.0	0.8–1.4
Temporary direct contacts between flocks†	No	230 (66)	66 (70)	1.0	–
	Yes	120 (34)	28 (30)	0.7	0.5–1.1
Stay of animals in other flocks with direct contacts	No	319 (91)	87 (93)	1.0	–
	Yes	31 (9)	7 (7)	0.7	0.4–1.3
Stay of animals from other flocks with direct contacts	No	332 (95)	91 (97)	1.0	–
	Yes	18 (5)	3 (3)	0.8	0.4–1.9
Presence of small ruminants in the vicinity of the farm	No	71 (20)	14 (15)	1.0	–
	Yes	279 (80)	80 (85)	1.1	0.7–1.7
Sharing paths	No	149 (43)	39 (41)	1.0	–
	Yes	201 (57)	55 (59)	0.9	0.7–1.2
Other indirect environmental contacts‡	No	311 (89)	85 (90)	1.0	–
	Yes	39 (11)	9 (10)	0.7	0.4–1.2
Feeding					
Purchase of raw materials§	No	213 (61)	66 (70)	1.0	–
	Yes	137 (39)	28 (30)	0.6	0.4– 0.8
Purchase of milk replacers¶	No	287 (82)	66 (70)	1.0	–
	Yes	63 (18)	28 (30)	2.0	1.4–2.7
Purchase of proprietary concentrates¶	No	79 (23)	7 (7)	1.0	–
	Yes	271 (77)	87 (93)	2.2	1.2–3.8
Purchase of milk replacers from factory 1	No	329 (94)	76 (81)	1.0	–
	Yes	21 (6)	18 (19)	3.1	2.1–4.6
Purchase of milk replacers from other factories	No	317 (91)	87 (93)	1.0	–
	Yes	33 (9)	7 (7)	0.9	0.5–1.6
Purchase of proprietary concentrates from factory 1	No	271 (77)	48 (51)	1.0	–
	Yes	79 (23)	46 (49)	2.6	1.9–3.5
Purchase of proprietary concentrates from factory 2	No	292 (83)	85 (90)	1.0	–
	Yes	58 (17)	9 (10)	0.4	0.2–0.7
Purchase of proprietary concentrates from other factories	No	228 (65)	54 (57)	1.0	–
	Yes	122 (35)	40 (43)	1.2	0.8–1.7
Other indirect contacts					
Artificial insemination	No	247 (71)	57 (61)	1.0	–
	Yes	103 (29)	37 (39)	1.0	0.7–1.5
Cesarean section performed by veterinarian	No	163 (47)	28 (30)	1.0	–
	Yes	187 (53)	66 (70)	1.9	1.3–2.7
Ear-tagging	No	236 (67)	55 (59)	1.0	–
	Yes	114 (33)	39 (41)	1.1	0.8–1.6
Sharing of farming devices	No	85 (24)	22 (23)	1.0	–
	Yes	265 (76)	72 (77)	0.7	0.5–1.0
Presence of dogs on the farm	No	325 (93)	84 (89)	1.0	–
	Yes	25 (7)	10 (11)	1.1	0.7–1.7
Purchase of hay	No	247 (71)	70 (74)	1.0	–
	Yes	103 (29)	24 (26)	0.7	0.5–1.0

*OR, odds ratio; CI, confidence interval. †Contacts by transhumance or common pastures with contacts between animals. ‡e.g., common pastures without direct contacts between animals. §Purchase of hay excluded because considered in other indirect contacts. ¶Independently of the factories.

### Data Collection

Information was collected by using a preestablished questionnaire to interview farmers and analyzing farm records. Questions related to potential risk factors covered the 4-year period preceding detection of the first clinical case of scrapie in case flocks and the 4-year period preceding the interview for controls. Additionally, information regarding potential confounding factors including flock size, production type (dairy, meat, or mixed), and intensification level of the flock production was recorded (Table 2). Interviews were conducted from May 1999 to July 2000 with 453 flock owners (98 cases and 355 controls). Nine flocks were excluded because they did not meet the inclusion criteria. A total of 444 flocks (94 cases and 350 controls) were included in the study. Data were encoded and then stored in an Access database (Microsoft Access 97 SR-2, Microsoft Corporation, Redmond, WA, USA).

**Table 2:** Multivariate analysis of potential confounding factors

Factors	Modalities*	No. controls (%)	No. cases (%)	Log-linear model	
				OR†	95% CI
Flock size	<133*	100 (29)	11 (12)	1.0	–
	133–236	87 (25)	24 (25)	2.5	1.1 – 5.5
	237–366	77 (22)	33 (35)	4.0	1.8 – 8.6
	>366	86 (25)	26 (28)	3.0	1.3 – 7.0
Type of flock	Dairy*	229 (65)	64 (68)	1.0	–
	Meat	113 (32)	27 (29)	1.0	0.3 – 3.2
	Mixed	8 (2)	3 (3)	1.2	0.3 – 5.5
Intensification criteria	None*	241 (69)	56 (60)	1.0	–
	Production monitoring	38 (11)	16 (17)	1.8	0.8 – 3.8
	Involvement in a breeding scheme	71 (20)	22 (23)	1.1	0.6 – 2.1

*Reference modality. †OR, odds ratio; CI, confidence interval.

### Study Sample

The flocks were mainly located in 2 departments (Pyrénées Atlantiques, n = 267/444, Aveyron n = 51/444). The others were widely distributed throughout metropolitan France. Ten mixed breeds and 23 pure breeds were included in the study. The flocks were mainly specialized in 1 type of production (66% in dairy production, 32% in meat production) (Table 2). The flock size ranged from 21 to 1,787 ewes (mean 274, SD 198).

### Analysis

Data analysis was conducted in 2 steps by using statistical models adjusted for the 2 matching factors through the corresponding cross-variable "strata" (main breed and location) treated as a stratification variable (13). First, to identify the confounding factors to be further analyzed (14), a log-linear model considered 5 factors, including flock size (number of ewes), production type, intensification level of the flock production as potential confounding factors, flock status, and strata. The model introduced the main effect of these 5 factors with all second interaction terms. Flock size was the only potential confounding factor notably associated with the flock status (Table 2). Second, to assess associations between flock status and risk factors, a generalized linear model for binary outcome was set up with the complementary log-log link function (Clog-log model) (14) (Appendix). This model considered the flock size by using the logarithm of the flock size as an offset (15,16). All exposures were considered as binary, and the absence of exposition was the reference modality for each risk factor. Factors notably associated with the flock status at 20% level through univariate analysis (Table 1) were selected for subsequent multivariate analyses. The univariate analysis consisted of the construction of a Clog-log model for each risk factor; strata were systematically introduced as covariate. Furthermore, 2 distinct multivariate models were applied to consider colinearity between feed type and feed factories in the foodborne risk study. The first model (multivariate Clog-log 1) analyzed feed types without regard to factories, whereas the second one (multivariate Clog-log 2) evaluated the risk according to the feed factories that produced milk replacers and proprietary concentrates. Regarding the proprietary feed factories, only the purchase of milk replacers and proprietary concentrates at factory 1 and the purchase of proprietary concentrates at factory 2 occurred frequently enough to be studied separately. Statistical software Splus (S-Plus 2000 Professional Release 2, Mathsoft, Inc., Seattle, WA, USA) was used to analyze the data.

## Results

According to the univariate analysis, 8 potential risk factors were selected (Table 1). Six risk factors were related to foodborne risk; the other 2 were related to purchasing ewes, and cesarean sections performed by the veterinarian. The subsequent multivariate model (multivariate Clog-log 1) (Table 3) showed a significant association between the flock status and using milk replacers. In addition, using the multivariate Clog-log 2 model milk replacers and proprietary concentrates from factory 1 were significantly associated with the flock status (Table 3).

**Table 3:** Multivariate analysis of risk factors

	Modalities*	No. cases (%)	No. controls (%)	Multivariate Clog-log 1		Multivariate Clog-log 2	
				OR†	95% CI	OR	95% CI
Direct contacts between flocks and indirect environmental contacts							
Purchase of ewes	No	39 (41)	123 (35)	1	–	1	–
	Yes			1.3	0.9–2.0	1.3	0.8–2.0
Feeding							
Purchase of raw materials (hay excluded)	No	28 (30)	137 (39)	1	–	1	–
	Yes			0.6	0.4–1.0	0.7	0.4–1.0
Purchase of milk replacers	No	28 (30)	63 (18)	1	–		
	Yes			1.9	1.2–3.0	NI	
Purchase of proprietary concentrates	No	87 (93)	271 (77)	1	–		
	Yes			1.5	0.7–3.4	NI	
Purchase of milk replacers from factory 1	No	18 (19)	21 (6)			1	–
	Yes			NI		1.9	1.0–3.5
Purchase of proprietary concentrates from factory 1	No	46 (49)	79 (23)			1	–
	Yes			NI		2.0	1.2–3.3
Purchase of proprietary concentrates from factory 2	No	9 (10)	58 (17)			1	–
	Yes			NI		0.7	0.3–1.5
Other indirect contacts							
Cesarean section performed by veterinarian	No	66 (70)	187 (53)	1	–	1	–
	Yes			1.6	0.9–2.8	1.4	0.8–2.5

*Reference modality = No. †OR, odds ratio; CI, confidence interval; NI, not in model.

## Discussion

The main finding of the study was the role of feed as a risk factor for scrapie. This is consistent with what has been shown for BSE in cattle. The use of proprietary concentrates, and more precisely the use of feeds containing meat and bone meal (MBM), was shown to have a major role in BSE infection of cattle (11). The agent of BSE is not inactivated by MBM processing methods, which were put into place by the industry in the late 1970s (17).

In France, MBM was authorized for small ruminants until July 1994. Moreover, the MBM ban proved to be <100% efficient; hundreds of BSE cases were observed in cattle in France born after the MBM ban of feeds for cattle. The exposure period that was investigated in the current study was from 1991 to June 2000, depending on the case. It occurred before the French MBM ban in feeds for all farmed animals in November 2000; furthermore, the period investigated was before the MBM ban for small ruminants in France for more than half of the cases. It is, therefore, plausible that sheep may have been contaminated by MBM in feeds throughout the 1990s, despite control measures. The results showed that 1 feed company was at risk for proprietary concentrates when others were not. This finding is in agreement with the fact that risk might depend on the type of raw materials used in the factory, as well as the way they were processed and used.

The risk attributable to milk replacers is the first evidence of such a TSE risk in animals. Milk replacers for all farmed species are made of skimmed cow milk enriched with vegetable or animal fats. Milk has not been shown to be at risk for scrapie transmission (18–20). Even if animal fat is not infectious, the animal fats that were incorporated in milk replacers may have been contaminated. Contamination could have occurred during collection at the slaughterhouse by contact with infectious material such as central nervous system or paravertebral ganglia. In France, these fats were prohibited for use in farm animal feeds in November 2000.

The same factory was identified as selling both the milk replacers and the proprietary concentrates at risk for scrapie. Most farmers buy both their feeds concentrates and milk replacers from the same wholesaler (which, in turn, buys from the same factory). Even if the effect of the 2 factors remained in the multivariate analysis, a confounding effect between these 2 factors cannot be excluded.

The main concern raised by this study is the nature of the infectious agent that was transmitted to sheep by means of feeds. It might be scrapie, but it could be also BSE, since cattle were infected by feeds during the same period in France. In 2005, BSE in a goat was first reported in France (21); in the United Kingdom, a goat that was thought to have scrapie in 1990 is being reexamined because it is now suspected to have had BSE (http://www.defra.gov.uk/news/2005/050208a.htm). In France, every index case animal from infected small ruminant flocks that has been reported since the surveillance began in 1990 has been biochemically tested to distinguish natural scrapie isolates from isolates sharing common biochemical features with experimental ovine BSE (validated by the TSEs Community Reference Laboratory of Weybridge, UK [unpub. data]). Among >400 small ruminant field isolates tested in France, only 1 isolate from a goat was indistinguishable from BSE. These arguments suggest that the agent transmitted to sheep by food was scrapie rather than BSE. Moreover, BSE is thought to have been transmitted and amplified by recycling contaminated carcasses into MBM on a regional basis (22). It follows that if the sheep identified as having scrapie did in fact have BSE, this misconception would have occurred in the same regions as BSE in cattle. That the areas of France most at risk for BSE in cattle (23) were different from those where scrapie occurred during the study does not suggest that the infectious agent for sheep was BSE.

Unexpectedly, the other hypotheses concerning the contamination of flocks with scrapie were not confirmed by the present study. In Norway, a matched case-control study showed 3 risk factors, though at a 10% a level: purchasing females, sharing rams, and sharing pastures between flocks (24). However, in a recent Irish study, purchasing breeding sheep through markets was not a risk factor for scrapie at a 5% a level (25). In the Norwegian study, feed did not appear to be a risk factor, whereas in the Irish study, feeding proprietary concentrates to lambs appeared to be protective. In the present study, purchasing ewes may not have emerged as a risk factor merely because of the lack of power of the study. The link between cesarean sections and scrapie occurrence that was observed in the univariate analysis was likely due to a confounding effect with the real risk factors and so became nonsignificant in the multivariate analyses.

Beyond the limits of the study, our results clearly show that in France, and more precisely in southwest France where most of the studied farms were located, the major risk for the introduction of scrapie in a flock during the 1990s was feeding certain proprietary concentrates and, possibly, milk replacers to sheep. Exposing sheep to TSE risk by feeding has certainly decreased since that time because of the complementary control measures taken in 1996 (ban on specified risk materials and cadavers in the processing of MBM) and 2000 (complete ban of MBM and certain animal fats for all farmed animals). However, it is essential to monitor these risk factors over time in France and to extend this kind of study to other countries in which the disease occurs.

The study results show strong evidence that TSEs can spread to sheep through feeding in field conditions, as is the case for cattle. Given the potential risk for humans, the possibility of BSE spreading to sheep must be taken seriously, even though the horizontal transmission of BSE in sheep would occur and stay at a low level (26), should such contamination occur (27). In any case, such findings support the need for a more comprehensive surveillance of TSEs in sheep, as well as the need to systematically examine all scrapie cases for their resemblance to BSE.

## Appendix

### Description of the Clog-log model

This appendix provides a description of the statistic model, a generalized linear model for binary outcome with the complementary log-log link function, used to assess the associations between flock status and risk factors. The construction is based on the probability (*P*) for a sheep flock to be qualified as an "infected scrapie flock," assuming independence and equiprobability for animals of a same flock to be infected by scrapie. This probability is equal to



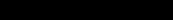



where *k* is the flock size and *p* the probability for an animal of the flock to be diagnosed infected by scrapie. Then, applying the complementary log-log function (Clog-log) on *P*, the quantity below was obtained.



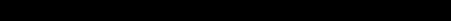



Therefore, the use of Clog-log as the link function (28) leads to model the probability to be infected at the animal level instead of at the flock level. In this model, flock size is introduced through the offset "Log(*k*)".



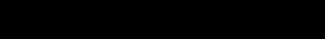



where Xj, j∈{1, …, q} is the vector of covariates. Moreover, if X_1_ and X_2_ are two exposure variables, then



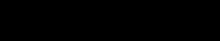



For values of *p* smaller than 10%, 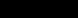 and 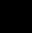 are very close. Hence, for small values of *p*,


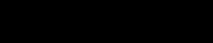
.

The parameters estimated from the complementary log-log model can be interpreted as those from a logistic model. The appropriate coding of exposure (X = 1) and nonexposure (X = 0) provides an easy interpretation of the parameters with OR = exp(β) and IC95% = [exp(β) +/- zα/2 s.e.(β)], zα/2 being issued from the cumulative distribution function of the standard normal distribution, and s.e. (β) being the standard error of parameter β (29,30).
